# Dynamic changes of miRNAs in skeletal muscle development at New Zealand rabbits

**DOI:** 10.1186/s12864-021-07896-5

**Published:** 2021-07-27

**Authors:** Jing Jing, Xichun Jiang, Cuiyun Zhu, Qi Zheng, Qianyun Ji, Huiqun Yin, Jingtong Huang, Yixiao Zhu, Jiao Wang, Shuaiqi Qin, Yinghui Ling

**Affiliations:** 1 College of Animal Science and Technology, Anhui Agricultural University, Anhui 230036 Hefei, People’s Republic of China; 2 Anhui Province Key Laboratory of Local Livestock and Poultry Genetic Resource Conservation and Bio- Breeding, Anhui Agricultural University, Anhui 230036 Hefei, People’s Republic of China; 3 Institute of Animal Science and Veterinary Medicine, Anhui Academy of Agricultural Sciences, Anhui 230031 Hefei, People’s Republic of China; 4 Reproductive Medicine Center, The 901st Hospital, Anhui 230031 Hefei, People’s Republic of China

**Keywords:** miRNA, Skeletal muscle, sRNA-seq, Rabbit, Development

## Abstract

**Background:**

miRNA is one of the crucial roles in the complex and dynamic network that regulates the development of skeletal muscle. The landscape of skeletal muscle miRNAs from fetus to adult in New Zealand rabbits has not been revealed yet.

**Results:**

In this study, nine RNA-seq libraries of fetus, child and adult rabbits’ leg muscles were constructed. A total of 278 differentially expressed miRNAs (DEmiRNAs) were identified. In the fetus vs. child group, the main functional enrichments were involved in membrane and transport. Pathway enriched terms of up-regulated DEmiRNAs were connected with the differentiation and hypertrophy of skeletal muscle, and down-regulated ones were related to muscle structure and metabolic capacity. In the child vs. adult group, functions were associated to positioning and transportation, and pathways were relevant to ECM, muscle structure and hypertrophy. Finally, ocu-miR-185-3p and ocu-miR-370-3p, which had the most target genes, were identified as hub-miRNAs in these two groups.

**Conclusions:**

In short, we summarized the highly expressed and uniquely expressed DEmiRNAs of fetus, child and adult rabbits’ leg muscles. Besides, the potential functional changes of miRNAs in two consecutive stages have been explored. Among them, the ocu-miR-185-3p and ocu-miR-370-3p with the most target genes were selected as hub-miRNAs. These data improved the understanding of the regulatory molecules of meat rabbit development, and provided a novel perspective for molecular breeding of meat rabbits.

**Supplementary Information:**

The online version contains supplementary material available at 10.1186/s12864-021-07896-5.

## Background

Rabbit meat is considered to be one of the healthiest meats and a functional food due to its excellent nutrition and dietary characteristics [1]. It contains high protein, essential amino acids and a variety of trace elements. Also, its sensitization and low cholesterol levels have also been demonstrated [1, 2]. Despite the intensification of rabbit farming and the advent of large-scale retailing, rabbit meat consumption is still mainly sold as loins and hind legs cuts. Consequently, the legs are one of the most commercially valuable parts [1]. Skeletal muscle is composed of muscle fibers, the contraction part of the muscle, connective tissue or extracellular matrix (ECM), and capillaries and nerves that provide blood to the muscle [3]. The type and characteristic of muscle fibers in skeletal muscle development affect the quality of meat [4]. Molecular biology and bioinformatics have become the basis for revealing the production performance of meat rabbits, which makes molecular breeding of domestic animals possible [5]. In recent years, the regulation of non-coding RNA (ncRNA), such as microRNA (miRNA), has also attracted considerable attention.

miRNAs are a class of small non-coding RNAs that play key roles in controlling skeletal muscle development [6]. In the past decade, the ability of miRNAs to bind the 3’-UTR of target mRNAs to perform biological functions has been widely accepted [7, 8]. For example, miR-206 played a key role in enhancing slow skeletal muscle and muscular dimorphism [9]. miR-1/133a mediated inhibition of Dlk1-Dio3 Mega gene cluster to complete the metabolic maturation of muscle stem cell differentiation [10]. Besides, miR-1 was also negatively correlated with HDAC4, which influenced the proliferation and differentiation of skeletal muscle satellite cells [11, 12]. Therefore, the potential functions of miRNAs in skeletal muscle development are worth exploring.

The potential miRNAs and their functions in rabbit skeletal muscles at different stages have not yet been elucidated. We herein hypothesized that the roles of the miRNAs in the skeletal muscles of rabbits at different stages from fetus to adults were changing. Thence, the miRNAs expression profiles in the leg skeletal muscles of fetus, child and adult New Zealand rabbits were explored in this study. Meanwhile, GO and KEGG were used to clarify the effect of miRNAs function conversion on development. Some potentially functional miRNA-mRNA networks have also been revealed. These screened data provided references for rabbit molecular breeding.

## Results

### Overview of rabbit leg muscle sRNA-seq (small RNA-seq) libraries

**Fig. 1:**
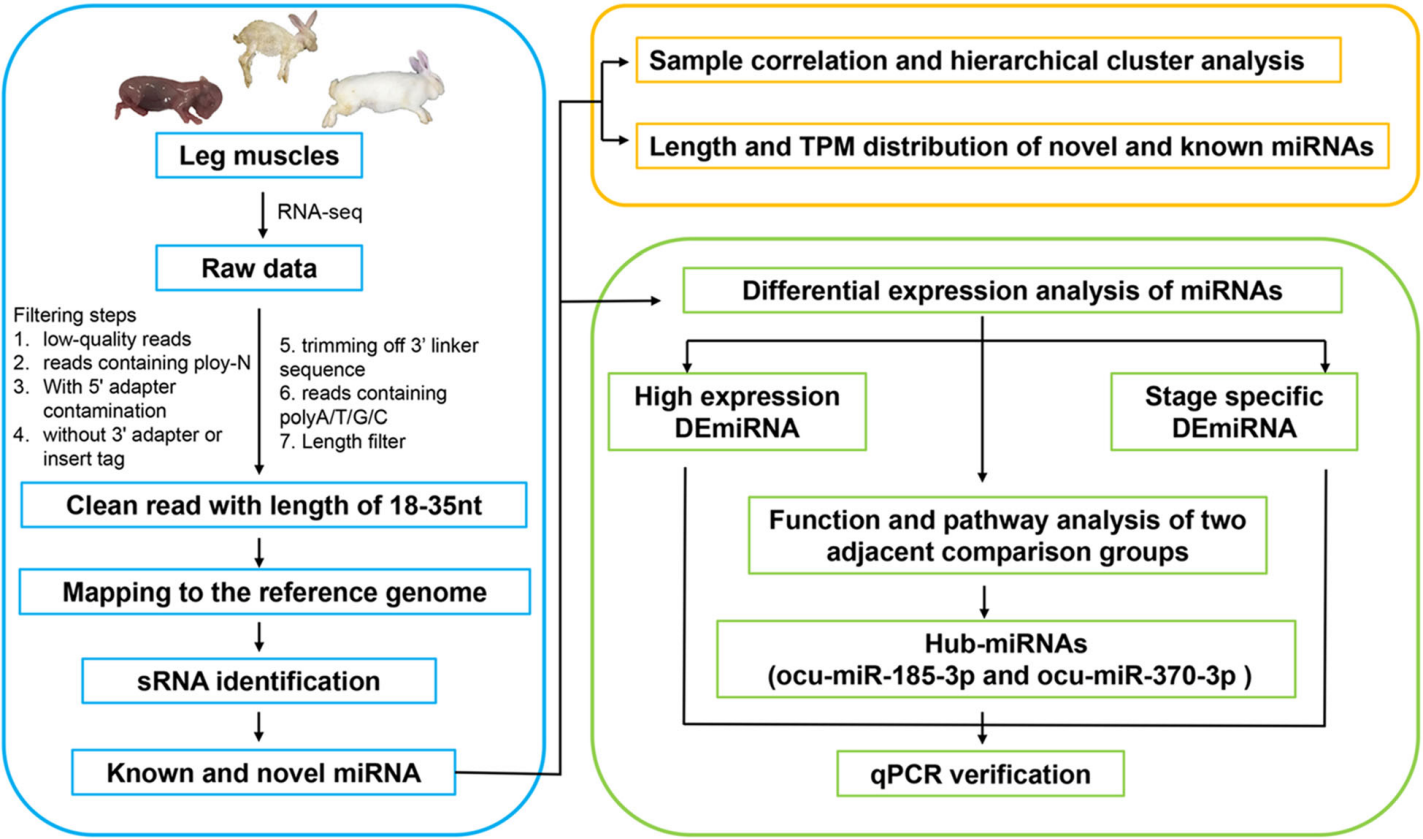
The method pipeline and experimental design to identify miRNAs. The content of the blue box is the process of sample collection, sequencing and miRNA identification. The content of the orange box is the analysis of data reliability. The content of the green box is differential analysis, screening and verification of miRNAs

**Fig. 2:**
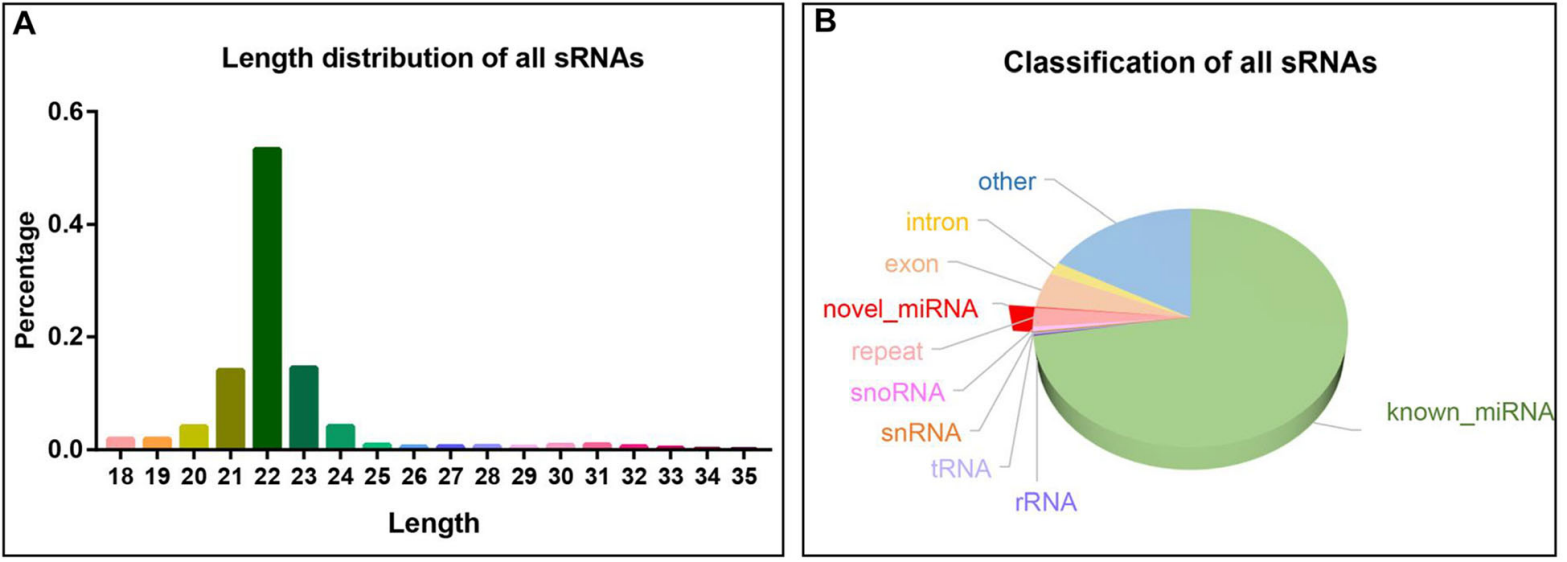
Length and identification of all sRNAs (small RNA). **A** Length distribution of all sRNAs. The abscissa is the length of sRNA (bp). The ordinate is the percentage of sRNA of a certain length. **B** Classification pie chart of all sRNAs. Different colors represent the proportion of different types of sRNA

Nine sRNA-seq libraries were constructed to screen the leg skeletal muscle miRNAs of New Zealand rabbits. The 9 libraries contain three stages: fetus, child and adult, with three replicates for each stage (Fig. 1). The 9 libraries identified raw reads ranging from 10.34 to 14.28 million with Q30 ranging from 96.96 to 97.71 % (Table S1). After libraries were filtered, clean reads ranging from 10.07 to 14.13 million were obtained (Table S1). Subsequent to length screening, 5.99 to 13.4 million clean reads were considered as sRNA reads, of which 94.12–96.76 % were mapped to the genome (Table S1). The lengths of all sRNAs were 18-35nt, most of which were concentrated in 21-23nt (Fig. 2A). All known miRNAs account for the largest proportion of sRNAs at about 72.5 % (Fig. 2B). Meanwhile, rRNA (Ribosomal RNA) accounted for only below 0.31 %, indicating that the data was reliable. Ultimately, 516 known miRNAs and 113 novel miRNAs were identified among all sRNAs (Table S2).

### Characterization of rabbit skeletal muscle miRNAs

**Fig. 3:**
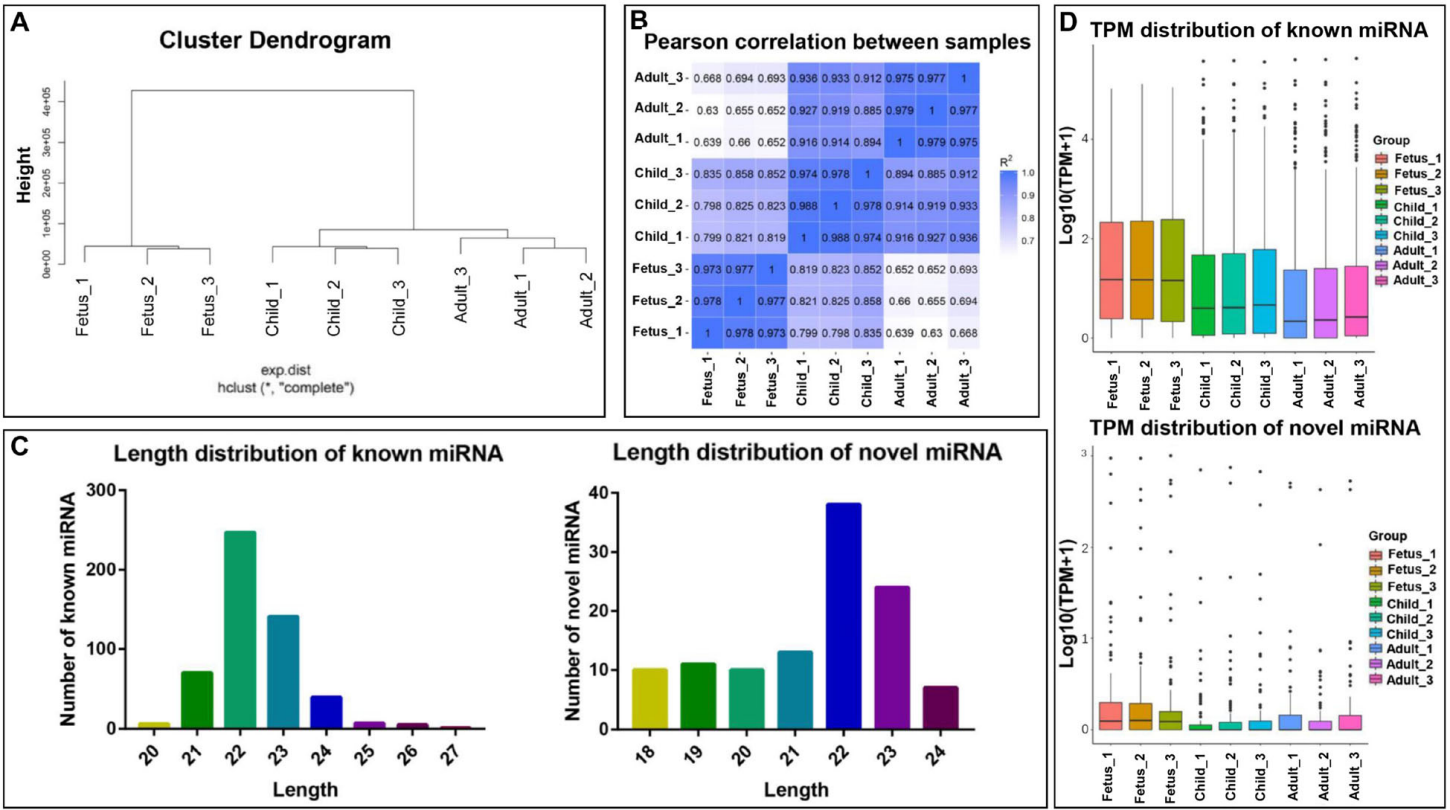
Clustering and characteristics of miRNAs. **A** Hierarchical cluster analysis (HCA) of all miRNAs. **B** Sample correlation analysis of all miRNAs. The color spectrum, ranging from white to blue, represents Pearson correlation coefficients ranging from 1 to 0, indicating high to low correlations. **C** Length distribution of known and novel miRNAs. The abscissa is the length of miRNA (nt). The ordinate is the number of known (left) and novel (right) miRNA of a certain length. **D** TPM distribution of known and novel miRNAs. The abscissa is the sample name. The ordinate is log_10_^(TPM+1)^. The box plot of each area corresponds to five statistics (from top to bottom are the maximum value, the upper quartile, the median value, the lower quartile and the minimum value)

In order to understand the identified miRNAs, we described the characteristics of all known and novel miRNAs. First, to verify the reliability of the data, all miRNAs were subjected to hierarchical cluster analysis (HCA) and sample correlation analyses. The HCA analysis also showed that the same stage also clustered together (Fig. 3A). The sample correlation showed that the three repetitions of each stage have the highest correlation, and the fetus stages were obviously different from the postnatal stages (Fig.3B). Then, the lengths of known and novel miRNAs were shown in the Fig. 3C. The lengths of the known mature miRNAs were between 20 and 27 nt, and the lengths of the novel miRNA were between 18 and 24 nt. Most miRNAs were concentrated at 22 and 23 nt (Fig. 3C). Next, the TPM distribution of miRNAs was explored. The distribution of known miRNAs at each stage was balanced, with similar distributions in child and adult stages (Fig. 3D). However, the TPM distribution of novel miRNAs at each stage was uniform.

### Differential expression analysis of miRNAs in rabbit skeletal muscle

**Fig. 4:**
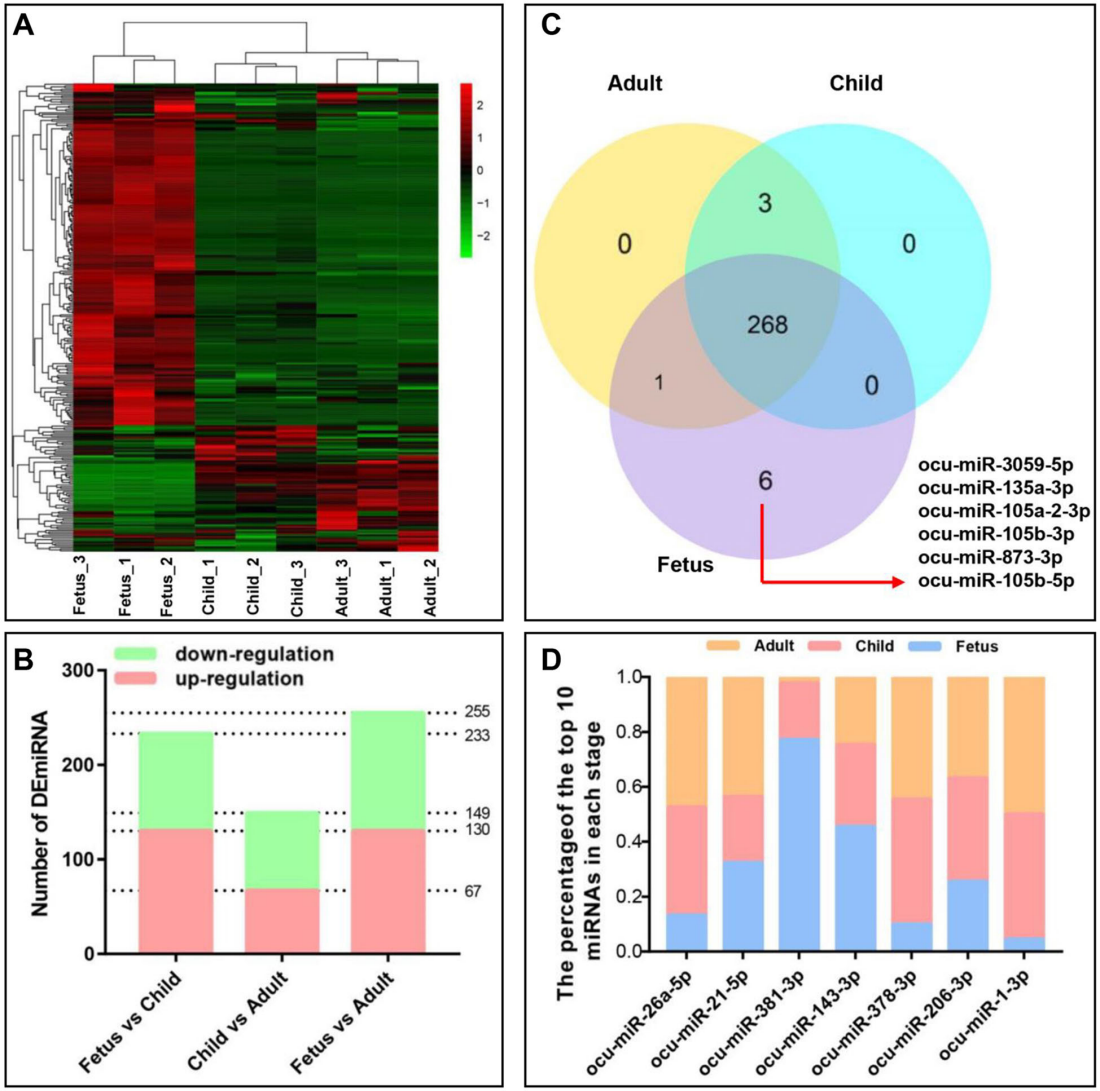
Identification and expression of differentially expressed miRNAs (DEmiRNAs). **A** Heat map and hierarchical clustering analysis of DEmiRNAs. Red, relatively high expression; green, relatively low expression. **B** The up- and down- regulated DEmiRNAs in different groups. **C** Venn diagram of DEmiRNAs at each stage. **D** The expression percentage of high expression DEmiRNAs at different stages. The abscissa is miRNA. The ordinate is the expression percentage of the corresponding miRNA in the three stages

So as to understand the regulation of miRNAs in prenatal and postnatal skeletal muscle, the differential expression analysis of miRNAs in fetus, child and adult rabbits’ skeletal muscles were performed. A total of 278 differential expressed miRNAs were identified at all stages, including 9 novel miRNAs and 269 known miRNAs (Table S3). The heat map clustering of DEmiRNAs indicated that the largest segmentation occurred between the fetus and child stages (Fig. 4A). Among them, 130 and 103 DEmiRNAs were up- and down-regulated in the fetus vs. child group; 67 and 82 DEmiRNAs were up- and down-regulated in the child vs. adult group; 130 and 125 DEmiRNAs were up- and down-regulated in the fetus vs. adult group (Fig. 4B). Interestingly, the 6 miRNAs were uniquely expressed at the fetus stage, including ocu-miR-3059-5p, ocu-miR-135a-3p, ocu-miR-105a-2-3p, ocu-miR-105b-3p, ocu-miR-873-3p and ocu-miR-105b-5p (Fig. 4C). In addition, the sum of the average expression of 7 DEmiRNAs was more than 100,000 TPM (Fig. 4D). These contained vital DEmiRNAs in the way of skeletal muscle development, such as miR-1, miR-206, miR-26a, etc. To further understand the role of DEmiRNAs in rabbit skeletal muscle development, target genes of DEmiRNAs in two adjacent stages were selected for Gene Ontology (GO) and Kyoto Encyclopedia and Genomes of Genes (KEGG).

### The roles of DEmiRNAs in fetus vs. child group

**Fig. 5:**
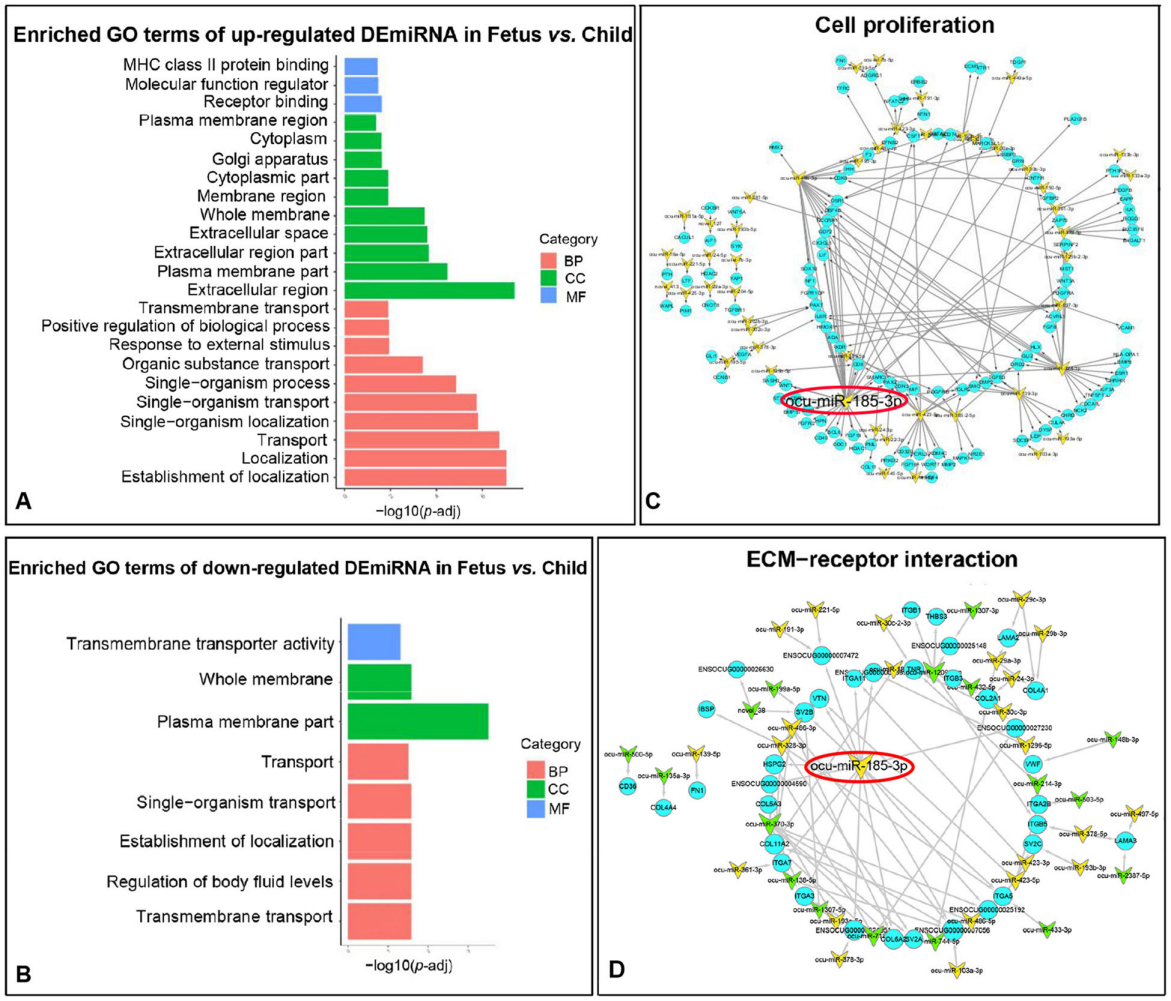
Function and pathway analysis of fetus vs. child group. **A-B **GO analysis of DEmiRNAs’ target genes in fetus vs. child group. Different colors represent different processes. BP: Biological process; CC: Cellular component; MF: Molecular function. **C** miRNA-mRNA network for cell proliferation. The yellow arrow represents up-regulated DEmiRNAs; the blue circle represents mRNAs. **D** miRNA-mRNA network for ECM-receptor interaction. The green arrow represents down-regulated DEmiRNAs

The target genes’ functions and pathways of the DEmiRNAs that were up- and down-regulated in the fetus vs. child group have been verified. The 130 up-regulated DEmiRNAs’ target genes were enriched in basic functions such as plasma membrane and material transport, and enriched in skeletal muscle development-related functions such as organelles, cell proliferation, and immune response (Fig. 5A, Table S4). The functions of 103 down-regulated DEmiRNAs in the fetus vs. child group were also related to the plasma membrane and material transport (Fig. 5B, Table S5). The number of muscle fibers is almost unchanged after birth, so muscle cell proliferation is the basis for the number of muscle fibers [13]. Therefore, we constructed a miRNA-mRNA network for “cell proliferation”. The 49 miRNAs involved in this function target 120 genes, of which ocu-miR-185-3p has 39 targeted genes and identified as a hub-miRNA for this function (Fig. 5C, Table S6).

Although all KEGG pathways were not significantly enriched (*P*-adj > 0.05), the data with *P* < 0.05 also showed some interesting results. Up-regulated DEmiRNAs’ target genes enriched in the key signal pathways of skeletal muscle differentiation and hypertrophy (*P* < 0.05), comprising MAPK signaling pathway, Notch signaling path-way and PI3K-Akt signaling pathway, etc. (Fig S1A, Table S4). At the same time, down-regulated DEmiRNAs were concerning tissue structure and metabolic capacity (Fig S1B, Table S5). Additionally, target genes of both up-regulated and down-regulated DEmiRNA were enriched in “ECM-receptor interaction”. In this pathway, 41 miRNAs targeted 36 genes, of which ocu-miR-185-3p also has the most target genes (Fig. 5D, Table S6). Therefore, ocu-miR-185-3p might exert an enormous function on this pathway.

### The role of DEmiRNAs in child vs. adult group

**Fig. 6:**
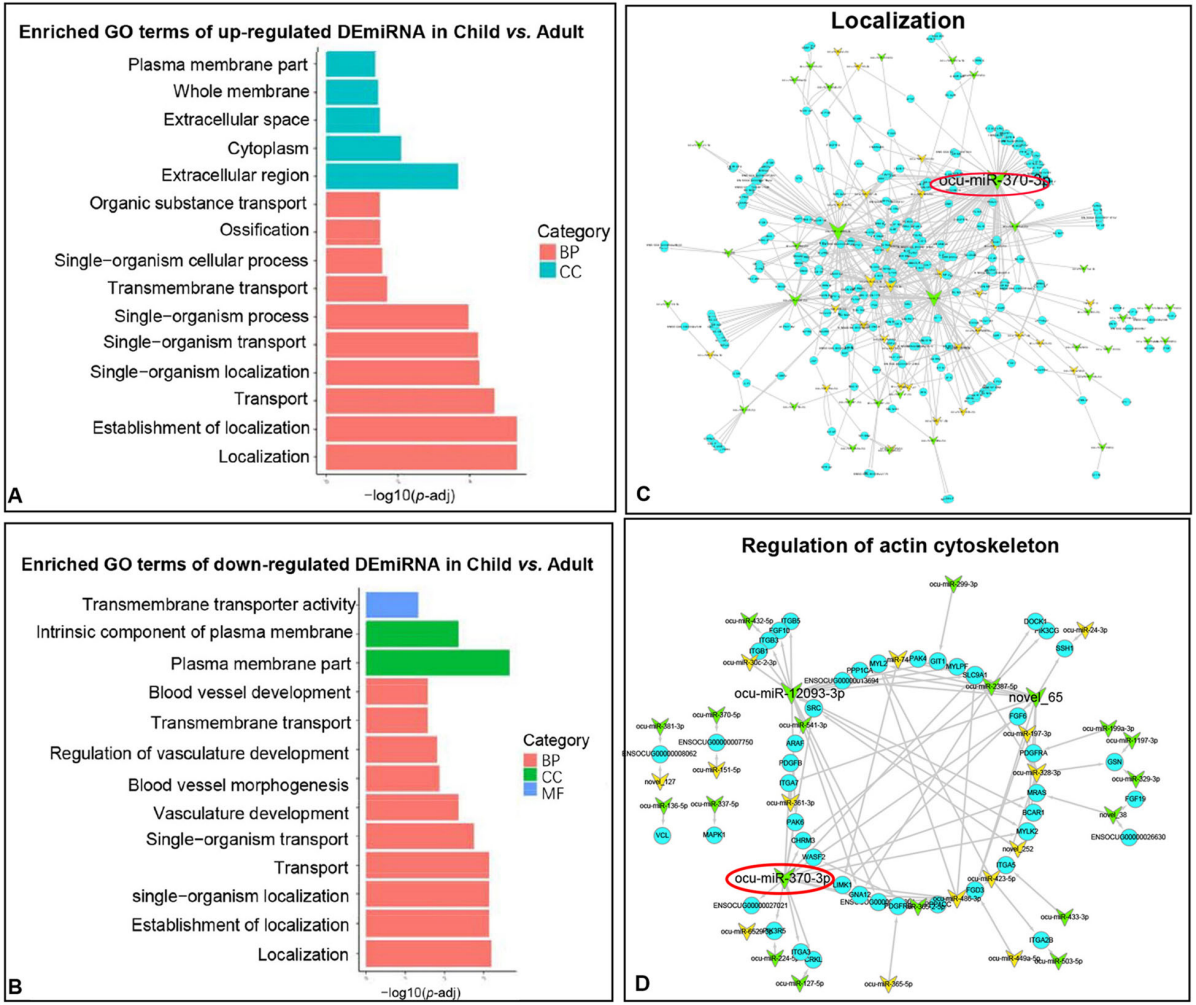
Function and pathway analysis of child vs. fetus comparison group. **A-B** GO analysis of DEmiRNAs’ target genes in child vs. fetus comparison group. Different colors represent different processes. BP: Biological process; CC: Cellular component; MF: Molecular function. **C **miRNA-mRNA network for localization. The yellow arrow represents up-regulated DEmiRNAs; the green arrow represents down-regulated DEmiRNAs; the blue circle represents mRNAs. **D** miRNA-mRNA network for regulation of actin cytoskeleton

The target genes of 67 up-regulated DEmiRNAs-enriched GO terms in the child vs. adult group were related to localization, plasma membrane and transport (Fig. 6A, Table S7). The target genes of 82 down-regulated DEmiRNAs in the child vs. adult group enriched GO terms related to localization, vascular development and protein transport (Fig. 6B, Table S8). Among them, “localization” was dynamically enriched in child vs. adult group, which was indispensable for the localization of substances and cellular components in skeletal muscle. The 260 genes involved in this function were targeted by 64 miRNAs, of which ocu-miR-370-3p targeted 99 miRNAs. Therefore, ocu-miR-370-3p was identified as hub-miRNA, which might play a significant effect on this function (Fig. 6C, Table S9).

Next, KEGG pathways with *P* < 0.05 were followed. Enriched terms of up-regulated DEmiRNAs showed that they were involved in the Regulation of actin cytoskeleton, MAPK signaling pathway and Linoleic acid metabolism and other pathways (Fig S2A, Table S7). Down-regulated DEmiRNAs’ target genes participated in skeletal muscle-related Focal adhesion, ECM-receptor interaction, Regulation of actin cytoskeleton and other pathways (Fig S2B, Table S8). “Regulation of actin cytoskeleton”, which related to skeletal muscle structure, was enriched in this group. The 45 genes involved in this pathway were targeted by 34 miRNAs, of which ocu-miR-12093-3p and ocu-miR-370-3p targeted 17 and 16 genes. This suggested that they might be the key factors for regulating the cytoskeleton of actin. At last, ocu-miR-370-3p was identified as hub-miRNA (Fig. 6D, Table S9).

### qPCR verification of DEmiRNAs in rabbit skeletal muscle

**Fig. 7:**
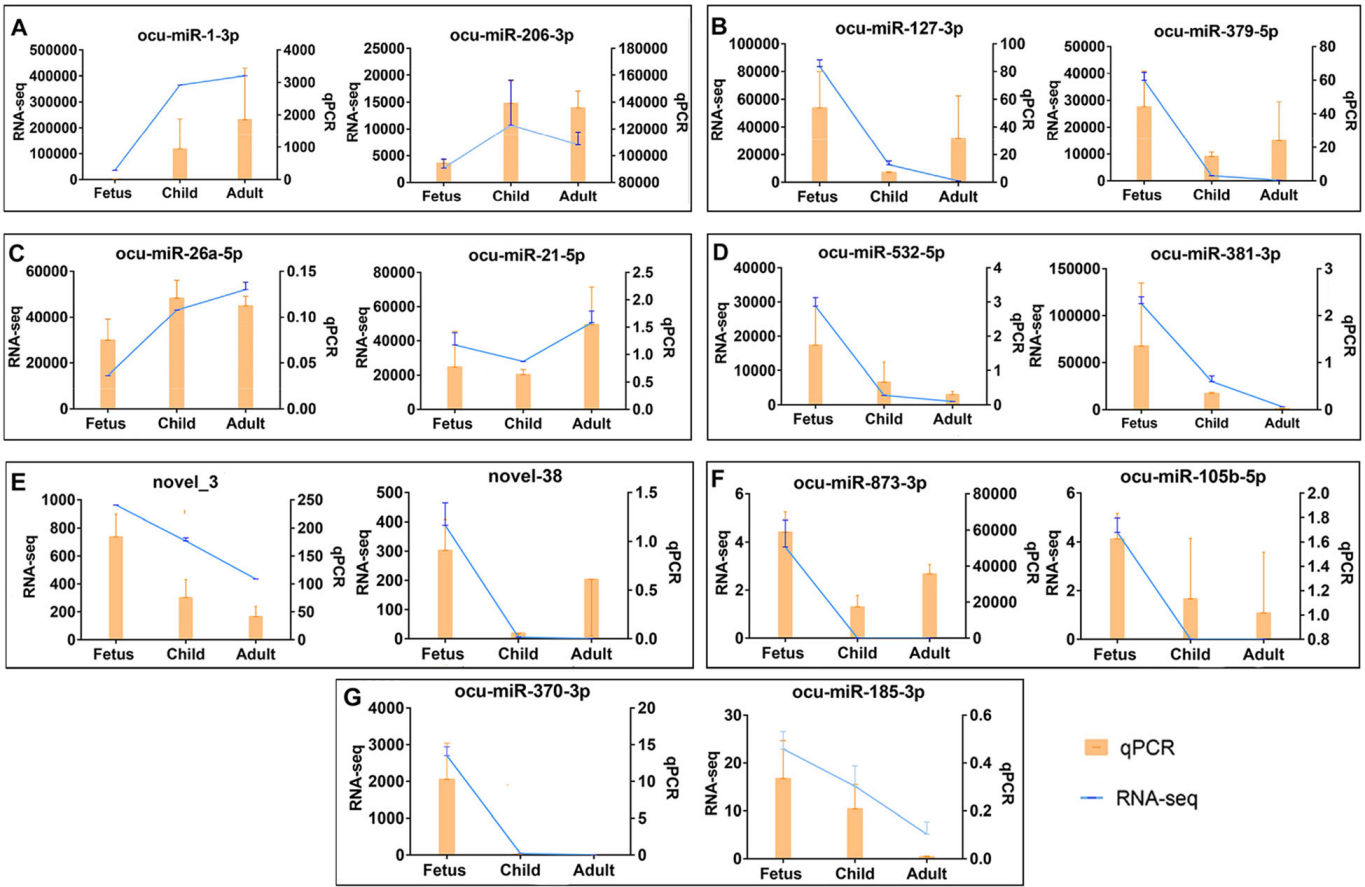
qPCR verification of DEmiRNAs. **A-G** qPCR (Bar chart, orange) and RNA-seq expression (Line chart, blue) validation of the indicated rabbit skeletal muscle miRNAs. **A-D** High expressed DEmiRNAs in the fetus vs. child (**A-B**) and child vs. adult (**C-D**) gourps. **E** Expression of novel DEmiRNAs, **F** High expressed only in fetus stage. **G** Expression of two hub-miRNAs

To verify the credibility of sRNA-seq, 14 DEmiRNAs were subjected to qPCR, comprising the highly expressed DEmiRNAs in the fetus vs. child (up-regulated DEmiRNAs: ocu-miR-206-3p and ocu-miR-1-3p; down-regulated DEmiRNAs:ocu-miR-379-5p and ocu-miR-127-3p) and child vs. adult (up-regulated DEmiRNAs: ocu-miR-21-5p and ocu-miR-26a-5p; down-regulated DEmiRNAs: ocu-miR-532-5p and ocu-miR-381-3p) groups, the highly expressed novel DEmiRNAs (novel_38 and novel_3), the highly expressed DEmiRNAs only in the fetal stage (ocu-miR-873-3p and ocu-miR-105b-5p), and two hub-DEmiRNAs (ocu-miR-370-3p and ocu-miR-183-3p). In addition, novel_38 and novel_3 were the two most expressed novel DEmiRNAs, and they target 110 and 12 genes, respectively. As expected, most DEmiRNAs expression trends were similar to sRNA-sEq. In the fetus vs. child group, ocu-miR-206-3p and ocu-miR-1-3p were up-regulated between fetus and child stage, ocu-miR-379-5p and ocu-miR-127-3p were down-regulated between fetus and child stage, respectively (Fig. 7A&B). In child vs. adult group, ocu-miR-21-5p was similar to data (Fig. 7C). However, the actual expression of ocu-miR-26a-5p, whose sRNA-seq data was up-regulated between the child and adult stages, slightly decreased in adulthood. Furthermore, ocu-miR-532-5p and ocu-miR-381-3p had a high expression among down-regulated DEmiRNAs in data (Fig. 7D). Meanwhile, 2 novel miRNAs (novel_38 and novel_3) with a downward trend in three stages have also been verified (Fig. 7E). Unexpectedly, sRNA-seq showed two DEmiRNAs (ocu-miR-873-3p and ocu-miR-105b-5p) that were uniquely expressed in the fetal stage, but the actual expression level showed a downward trend instead of unique expression (Fig. 7F). Finally, the expression of the two hub-miRNAs (ocu-miR-370-3p and ocu-miR-183-3p) also showed the same trend as the data (Fig. 7G).

## Discussion

The main goal of meat animal breeding in agriculture is to produce as much high-quality protein food source as possible with the least input. In this study, the differential expression profiles of miRNAs in the skeletal muscle of rabbit fetus, child and adult stages were obtained. In the fetus vs. child group, the main enriched membrane and transport were changing dynamically. In the pathway, up-regulated DEmiRNAs were related to skeletal muscle differentiation and hyper-trophy, and down-regulated DEmiRNAs had a bearing on tissue structure and metabolic capacity. In the child vs. adult group, the functions were associated with localization and transport, pathways were related to ECM, muscle structure and hypertrophy. Among them, the ocu-miR-185-3p and ocu-miR-370-3p with the most target genes were selected as hub-miRNAs. These results verify the hypothesis that the potential role of miRNAs in rabbit skeletal muscle development is changing.

Muscles have an incredible ability to change their functional characteristics in order to adapt and function optimally in response to the physiological state of the animal (e.g., growing, maintaining, or senescing). The regulatory non-coding RNAs at different stages of muscle development in multiple species are gradually being revealed, such as duck [14], sheep [15], pig [16] and goat [17]. For example, 1,498 circRNAs were identified in the skeletal muscles of pigs at three stages after birth. Among them, the continuous stage and unique expression differential circRNAs and their potential functions have also been explored [16]. In addition, the dynamic expressions of miRNAs and lncRNAs in 7 stages of goat was revealed, which have the potential to affect skeletal muscle development [17, 18]. Therefore, exploring the regulatory molecules of muscle physiological changes at different stages is equally essential for the breeding of meat rabbits. The top 10 DEmiRNAs expressed in the 7 stages of the *longissimus dorsi* muscle of goats include chi-miR-1, chi-miR-206, chi-miR-148a-3p, chi-miR-381, chi-miR-127-3p, chi-let-7i-5p, chi-miR-26a-5p, chi-miR-10b-5p, chi-miR-378-3p, chi-let-7f-5p. Among them, 5 DEmiRNAs (ocu-miR-1, ocu-miR-206, ocu-miR-381, ocu-miR-26a-5p, ocu-miR-378-3p) were also highly expressed in rabbits. However, the top 10 DEmiRNAs in the 5 stages of pigs from birth to 7 years old were miR-378-1/-2-3p, miR-127-3p, miR-191-5p, miR-486-2-5p, miR-143-3p, miR-10a-5p, miR-148a-3p, miR-99a-5p, miR-30e-5p and miR-199a-1/-2-5p [19]. miR-378-3p was highly expressed in the skeletal muscles of goat, pigs and rabbits. Except for miR-378-3p, the highly expressed DEmiRNAs of pigs and rabbits were only miR-143-3p. This study explored the function of DEmiRNAs and selected hub-miRNAs in the leg skeletal muscles of rabbits at the fetus, child and adult stages.

All the identified 516 known miRNAs and 113 novel miRNAs were concentrated on 22 and 23 nt in length. The sample correlation and HCA verified the reliability of the samples. From these miRNAs, we obtained 9 novel DEmiRNAs and 269 known DEmiRNAs, and performed functional analysis on them. Among them, 7 DEmiRNAs have a TPM greater than 100,000, comprising ocu-miR-1-3p, ocu-miR-206-3p, ocu-miR-378-3p, ocu-miR-143-3p, ocu-miR-381-3p, ocu-miR-21-5p, ocu-miR-26a-5p. Most of them have been verified to have an indispensable effect on development of skeletal muscle. In addition to miR-1 and miR-206, miRNA-143 targets Igfbp5 to mediate senescence of muscle stem cells, thereby affecting myogenesis [20]. miR-21, a negative regulator of PTEN expression, leads to changes in Akt activity, consistent with changes in insulin sensitivity in muscles [21]. These findings suggested that miR-378-3p and miR-26a-5p, which have not been studied in skeletal muscle development, may also have necessary roles. In addition, novel_3 and novel_38 were the top 2 expressed novel DEmiRNAs, which targeted 12 and 110 genes, respectively. The target genes CaMKK2 [22], CD9 [23], BAG3 [24]and NUAK2 [25] of novel_3 are related to muscle regeneration. Target gene of novel_38 were not enriched in GO, but the target gene STAT5B was involved in the growth hormone and JAK-STAT signaling pathways, which were associated with skeletal muscle development [26, 27]. Furthermore, it was interesting that there were 6 miRNAs that uniquely expressed in the fetus stage, which might have unique roles. Moreover, DEmiRNAs might have distinctive regulatory effects on development at different stages, so the potential roles of DEmiRNAs in two consecutive stages of skeletal muscle have been explored.

In the fetus vs. child group, the all DEmiRNAs were involved in various pathways and functions related to skeletal muscle development. For instance, 124 genes, targeted by up-regulated 49 DEmiRNAs, were involved in the function of cell proliferation. Among them, ocu-miR-302b-3p, ocu-miR-302c-3p, ocu-miR-486-3p, and ocu-miR-185-3p targeted PAX7, which was a marker of muscle stem cell that promotes proliferation and commitment to the myogenic lineage and inhibited the genes driving differentiation [28]. ECM-receptor interaction, which was enriched in both up-regulation and down-regulation, was principal part of skeletal muscle and plays a major role in the force transmission, maintenance and repair of muscle fibers (Figure S3) [3]. Ocu-miR-185-3p had the most targeted genes in “cell proliferation” and “ECM-receptor interaction “, so it was selected as the hub-miRNA for this function and pathway. The relationship between ocu-miR-183-3p and skeletal muscle has not been studied, but it acts as a sponge of circHIPK3 to regulate myocardial atrophy [29]. These target genes of ocu-miR-183-3p included FGF19 (It regulated skeletal muscle mass and protect muscle from atrophy by increasing muscle fiber size) [30], FGF2 (It was an important stimulator of satellite cells) [31], and WNT1 (It was a key factor in the canonical Wnt signaling pathway that is indispensable for muscle proliferation) [32] and so on.

In the child vs. adult group, the enriched functions of up-regulated and down-regulated DEmiRNAs were related to localization and transport. “Localization” is indispensable for the localization of substances and cellular components in skeletal muscle. For example, MYOF, targeted by ocu-miR-365-2-5p, regulates the localization and accumulation of IGF receptors in myoblasts and necessary for muscle growth [33]. Meanwhile, these genes also contain interesting pathways related to skeletal muscle such as “Regulation of actin cytoskeleton” (Figure S4). “Actin cytoskeleton” is the basis of muscle cell contraction and also participates in the asymmetric division of muscle stem cells during proliferation [34, 35]. Ocu-miR-370-3p was considered as hub-miRNA because its large number of target genes contained in “Regulation of actin cytoskeleton” and “localization”. miR-370-3p inhibits the transition of fast muscle fibers [36], and it promotes the proliferation and cell cycle of vascular smooth muscle cells [37]. The genes of ocu-miR-370-3p comprised CRKL (it was noncatalytic adaptor proteins necessary for the formation of neuromuscular synapses which function downstream of muscle-specific kinase) [38], FGF6 (it reduced skeletal muscle atrophy by relying on the ERK1/2 mechanism and enhanced the conversion of slow muscle to fast muscle fibers) [39], TPM1 (it had  an effect on the formation of fast muscle or slow muscle) [40]. Furthermore, the target genes of up-regulated DEmiRNAs were also enriched in the MAPK signaling pathway of muscle hypertrophy [41] MRAS targeted by ocu-miR-328-3p, was a component of RAS, which was the main down-stream effector of the classic MAPK signaling pathway [42]. MAPKAPK2/3, targeted by ocu-miR-328-3p, ocu-miR-486-3p, ocu-miR-423-5p, was a protein kinase downstream of the MAPK signaling pathway. And its enzyme activity was the considerable factor for regulating striated muscle function [43]. These data clarified the potential roles of DEmiRNAs at different stages, and selected ocu-miR-185-3p and ocu-miR-370-3p as hub-miRNAs in the two comparison groups.

## Conclusions

In this study, sRNA-seq was used to construct a miRNA libraries of rabbit leg skeletal muscle in fetus, child and adult stages. Firstly, we summarized the highly expressed and uniquely expressed DEmiRNAs. Besides, the potential functional changes of miRNAs in two consecutive stages have also been explored. Among them, the ocu-miR-185-3p and ocu-miR-370-3p with the most target genes were selected as hub-miRNAs. These results provided a better understanding of the role of miRNAs in skeletal muscle development of meat rabbits and a novel insight into molecular breeding of meat rabbits.

## Materials and methods

### Sample collection

The leg muscles of this project were collected from fetus, child and adult New Zealand rabbits which were maintained with a unified management system. During this period, the rabbit house was kept dry and clean. Child (6-week-old, 0.86 ± 0.083 kg, *n* = 3) and adult (6-month-old with 2 weeks gestation, 4.37 ± 0.033 kg, *n* = 3) New Zealand rabbits were euthanized by air injection into the ear vein after anesthesia. Two-week-old fetuses’ rabbits were taken from 6-month-old adult rabbits. A total of 3 fetuses’ rabbits (2-week-old, 9.14 ± 0. 33 g) were selected, and the mother of each fetus was different. All rabbits’ left leg muscles were collected as samples. All collected leg skeletal muscles were rinsed with PBS containing 100× penicillin and streptomycin 3 times. Subsequently, the samples in the cryopreservation tubes were immediately put into liquid nitrogen and transferred to the − 80℃ refrigerator after 24 h.

### Small RNA libraries construction and sequencing

RNA was extracted with animal total RNA isolation kit (Foregene co., ltd., Chengdu, China). In addition, the degradation, purity, concentration and integrity of RNA were checked by 1 % agarose gel electrophoresis, NanoPhotometer® spectro-photometer (IMPLEN, CA, USA), and RNA Nano 6000 Assay Kit of the Agilent Bio-analyzer 2100 system (Agilent Technologies, CA, USA), respectively.

A total of 3 µg RNA per sample was used as input material for library construction. Then the libraries were constructed by NEBNext®Multiplex Small RNA Samples Prep Kit Set for Illumina® (NEB, MA USA). In short, based on the special structure of the small RNA 3’ and 5’ ends (the 5’ end has a complete phosphate group and the 3’ end had a hydroxyl group), the adaptor was directly added to the small RNA 3’ and 5’. Then first-strand cDNA was synthesized through M-MuLV Reverse Transcriptase (RNase H–). PCR amplification was performed using LongAmp Taq 2X Master Mix, SR Primer for Illumina and index (X) primer. Subsequently, the PCR products were subjected to 8 % PAGE gel electrophoresis to separate the target DNA fragments of 140 ~ 160 bp, the products recovered by the gel cutting were the cDNA libraries. After the library of each sample was constructed, it was initially quantified by Qubit® RNA Assay Kit in Qubit® 2.0 Flurometer (LifeTechnologies, CA, USA). Subsequently, the insert size of the library was detected with Agilent Bioanalyzer 2100 system. Finally, the different libraries were pooled according to the effective concentration and targeted offline data requirements, and then sequenced by Illumina SE50. The clustering of the index-coded samples was performed on a cBot Cluster Generation System using TruSeq SR Cluster Kit v3-cBot-HS (Illumia) according to the manufacturer’s instructions. The libraries’ preparations were sequenced on the Illumina Hiseq 2500/2000 platform.

### Data filtering and quality assessment

The raw data in fastq format was first processed by custom Perl and python scripts. The original data files have been uploaded and published to the NCBI SRA database. The accession number is PRJNA692651. The name is “miRNA raw data of rabbits’ hid leg skeletal muscle in fetus, child and adult”. At the same time, Q20, Q30, and GC-content of the raw data were calculated. In order to ensure the quality of analysis, raw reads were processed to obtain clean reads. The entire filtering steps include removing low-quality reads (reads with a quality value of sQ ≤ 20 that account for more than 30 % of the entire reads), reads containing ploy-N, reads with 5’ adapter contamination, reads without 3’ adapter or insert tag, trimming off 3’ linker sequence, reads containing poly A/T/G/C (most poly A/T/G/C, may be due to sequencing errors). Then, the 18 ~ 35 nt fragments in the clean reads were screened for all downstream analysis.

### Identifications of sRNA

Through bowtie [44], the length-screened sRNA was located on the reference sequence of rabbit (*Oryctolagus cuniculus*, Genome assembly: OryCun2.0 GCA_000003625.1). The distribution of sRNAs without mismatch on the reference sequences were analyzed. To make every unique sRNA mapped to only one annotation, we followed the following priority rule: known miRNA > rRNA > tRNA (Transfer RNA, tRNA) > snRNA (Small nuclear RNA) > snoRNA (Small nucleolar RNA) > repeat > gene > NAT-siRNA (Natural antisense transcript - small interfering RNA) > gene > novel miRNA > ta-siRNA (Trans-acting siRNA). The total rRNA proportion was used a marker as sample quality indicator.

The reads mapped to the reference sequence were aligned with the specified range sequence in miRBase20.0, and mirdeep2 [45] and srna-tools-cli were used to obtain the potential miRNAs and draw the secondary structures. Custom scripts were used to obtain the miRNAs’ counts as well as base bias on the first position of identified miRNAs with a certain length and on each position of all identified miRNAs respectively. To remove tags originating from protein-coding genes, repeat sequences, rRNA, tRNA, snRNA, and snoRNA, small RNA tags were mapped to RepeatMasker and Rfam database from the specified species. Finally, the signature hairpin structure of the miRNAs’ precursors was used to predict novel miRNAs. The miRNA collections, predicted by miRNA prediction software miREvo [46] and mirdeep2 [45], were identified as novel miRNAs in rabbits.

### Expression, target genes and difference analysis of miRNAs

The expression of known and novel miRNAs in each sample was counted, and the expression was normalized with transcripts per million (TPM) [47]. Then, the target genes of miRNAs were performed by miRanda [48]. Subsequently, differential expression analysis of two stages was performed using the DESeq R package (3.0.3). The *P* values was adjusted using the Benjamini & Hochberg method. Corrected *P* value (*P*-adj) of 0.05 was set as the threshold for significantly differential expression.

### Function and pathway analyses of target genes

GO enrichment analysis was performed on the target gene candidates of the DEmiRNAs by GOseq [49]. KOBAS software was used to test the enrichment of target gene candidates in the KEGG pathway.

### Quantitative PCR

Reverse transcription of selected miRNAs by the GoScrip Reverse Transcriptase kit (Promega, Madison, WI, USA) according to the manufacturer’s Guide. All of the primer pairs (Table S10) were designed using miRperimer and synthesized by the synthesized by the TsingKe biological technology company. The U6 housekeeping gene was amplified as a control. Next, GoTaq qPCR master mix (Aidlab, catalog number: APC6102) was used to perform qPCR on the LightCycler 96 (ABI) real-time PCR instrument. Each stage has 3 biological replicates. The quantities of miRNAs were normalized to the U6 and calculated as 2^−ΔΔCt^. One-way ANOVA analysis of the normalized data was then conducted using SPSS version 19.0 for Windows. Duncan’s multiple comparisons were performed for testing significant differences between mean values at different stages.

## Supplementary Information

**Additional file 1: Figure S1. ** Pathway analysis of the fetus *vs. *child group. (A) All enriched pathways of up-regulated DEmiRNAs’ target genes. (B) All enriched pathways of down-regulated DEmiRNAs’ target genes.

**Additional file 2: Figure S2. ** Pathway analysis of the child* vs.* adult group. (A) All enriched pathways of up-regulated DEmiRNAs’ target genes. (B) All enriched pathways of down-regulated DEmiRNAs’ target genes.

**Additional file 3: Figure S3. **Details of DEmiRNAs’ target genes involved in ECM-receptor interaction in the fetus *vs.*child group. (A) Target genes of up-regulated DEmiRNAs. (B) Target genes of down-regulated DEmiRNAs.

**Additional file 4: Figure S4. **Details of DEmiRNAs’ target genes involved in regulation of actin cytoskeleton in the child *vs. *adult group. (A) Target genes of up-regulated DEmiRNAs. (B) Target genes of down-regulated DEmiRNAs.

**Additional file 5: Table S1.**The overview of RNA-seq data.

**Additional file 6: Table S2.**All identified miRNAs and their expression levels.

**Additional file 7: Table S3:**All identified DEmiRNAs. Sheet 1 ("TPM"): All DEmiRNAs and their expression levels. Sheet 2 ("Average"): The average expression and total expression of all DEmiRNAs. Sheet 3-5: DEmiRNAs identified in different groups.

**Additional file 8: Table S4.**The GO and KEGG analyses of up-regulated DEmiRNAs in fetus *vs.* child group.

**Additional file 9: Table S5. **The GO and KEGG analyses of down-regulated DEmiRNAs in fetus* vs.* child group.

**Additional file 10: Table S6:**The GO and KEGG analyses of up-regulated DEmiRNAs in child *vs. *adult group.

**Additional file 11: Table S7.**The GO and KEGG analyses of down-regulated DEmiRNAs in child *vs.* adult group.

**Additional file 12: Table S8.** The primers of reference gene and miRNAs.

**Additional file 13: Table S9.** miRNA-mRNA network for localization (sheet 1) and regulation of actin cytoskeleton (sheet 2).

**Additional file 14: Table S10.** The primers of reference gene and miRNAs.

## Data Availability

The original data files have been uploaded and published to the NCBI SRA database. The accession number is PRJNA692651, the name is "miRNA raw data of rabbits' hid leg skeletal muscle in fetal, child and adult" (https://www.ncbi.nlm.nih.gov/sra?LinkName=biosample_sra&from_uid=16955474).

## Abbreviations

miRNA: microRNA
DEmiRNA: Differential expressed miRNA
GO: Gene Ontology
KEGG: Kyoto Encyclopedia of Genes and Genomes
HCA: Hierarchical cluster analysis
qPCR: Quantitative PCR
sRNA: Small RNA
rRNA: Ribosomal RNA
tRNA: Transfer RNA
snRNA: Small nuclear RNA
snoRNA: Small nucleolar RNA
NAT-siRNA: Natural antisense transcript - small interfering RNA
ta-siRNA: Trans-acting siRNA
