# Supplementary figures and images for: Dynamic changes of miRNAs in skeletal muscle development at New Zealand rabbits
Source: BMC Genomics. 2021 Jul 27;22:577. doi: 10.1186/s12864-021-07896-5 (PMC8314457; doi:10.1186/s12864-021-07896-5)

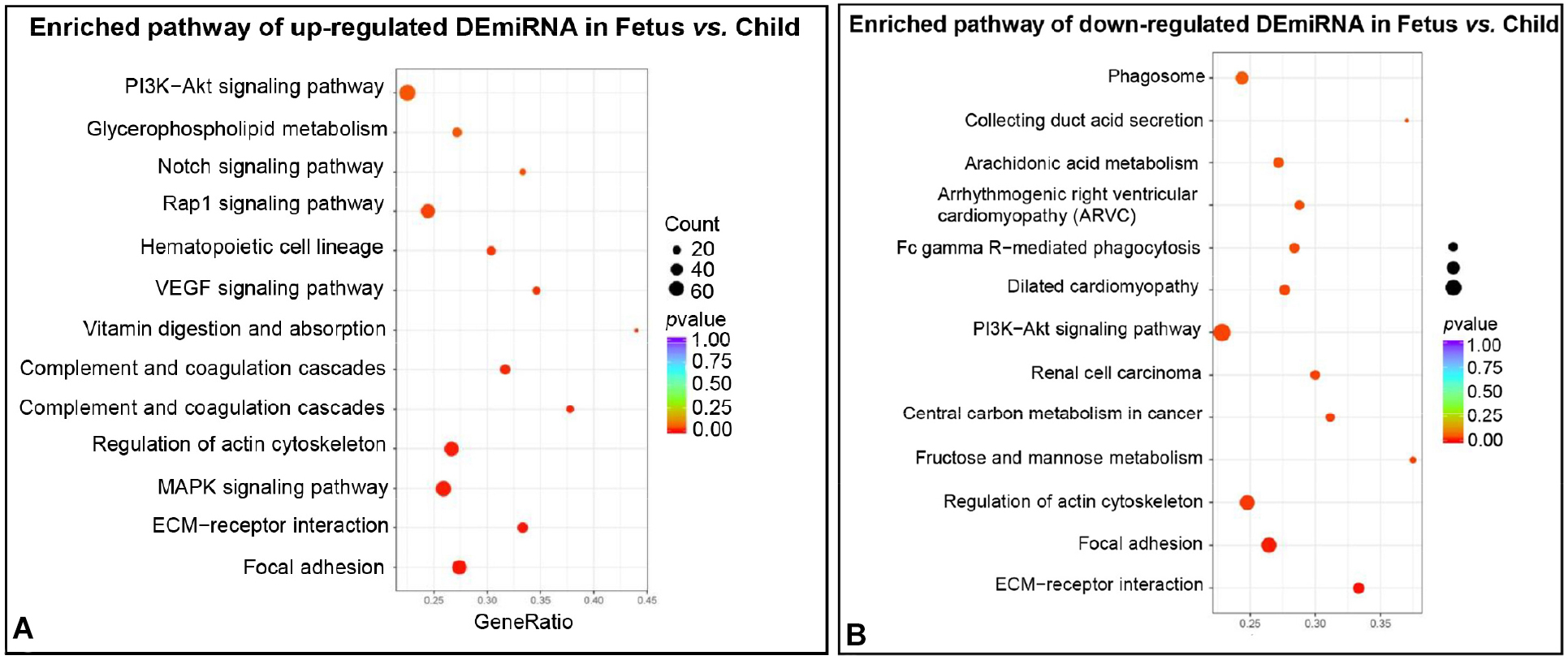

Supplement: Supplementary file 1 — Additional file 1: Figure S1. Pathway analysis of the fetus vs. child group. (A) All enriched pathways of up-regulated DEmiRNAs’ target genes. (B) All enriched pathways of down-regulated DEmiRNAs’ target genes. [file 12864_2021_7896_MOESM1_ESM.jpg]

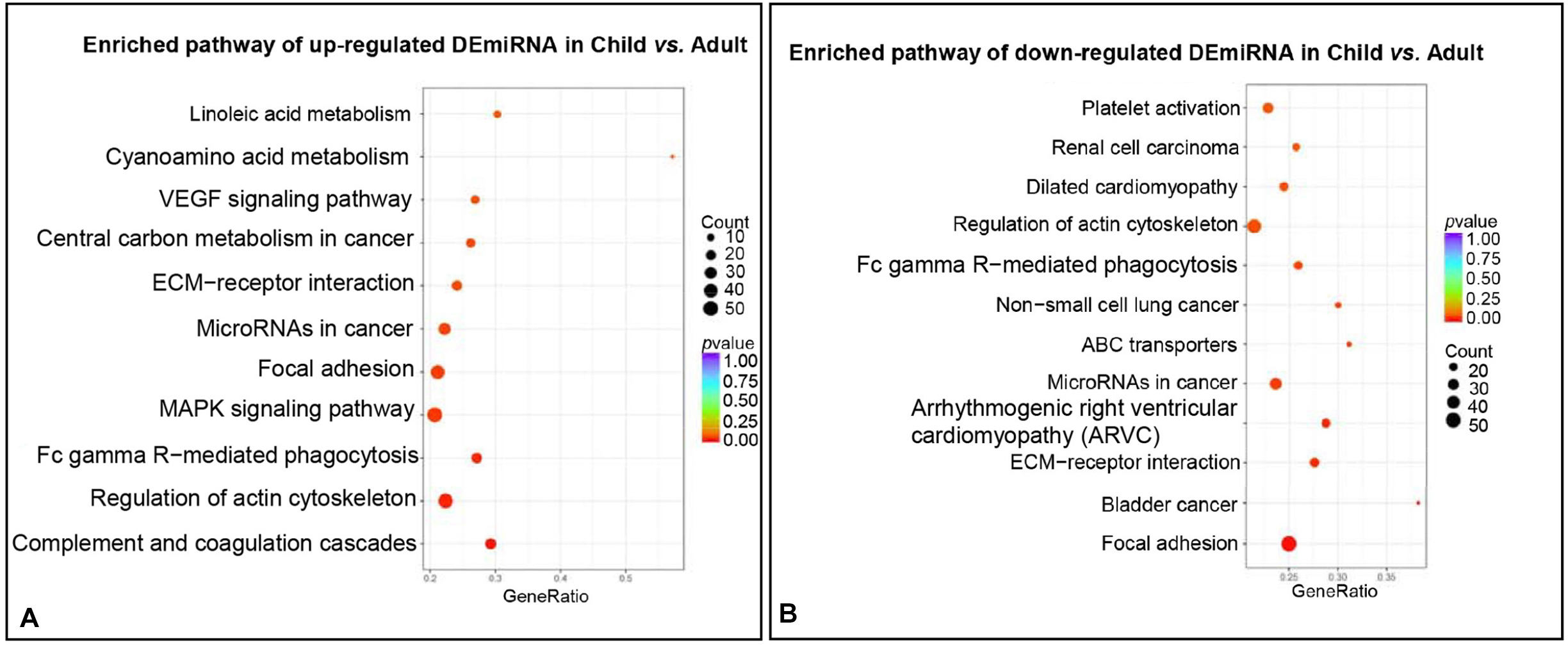

Supplement: Supplementary file 2 — Additional file 2: Figure S2. Pathway analysis of the child vs. adult group. (A) All enriched pathways of up-regulated DEmiRNAs’ target genes. (B) All enriched pathways of down-regulated DEmiRNAs’ target genes. [file 12864_2021_7896_MOESM2_ESM.jpg]

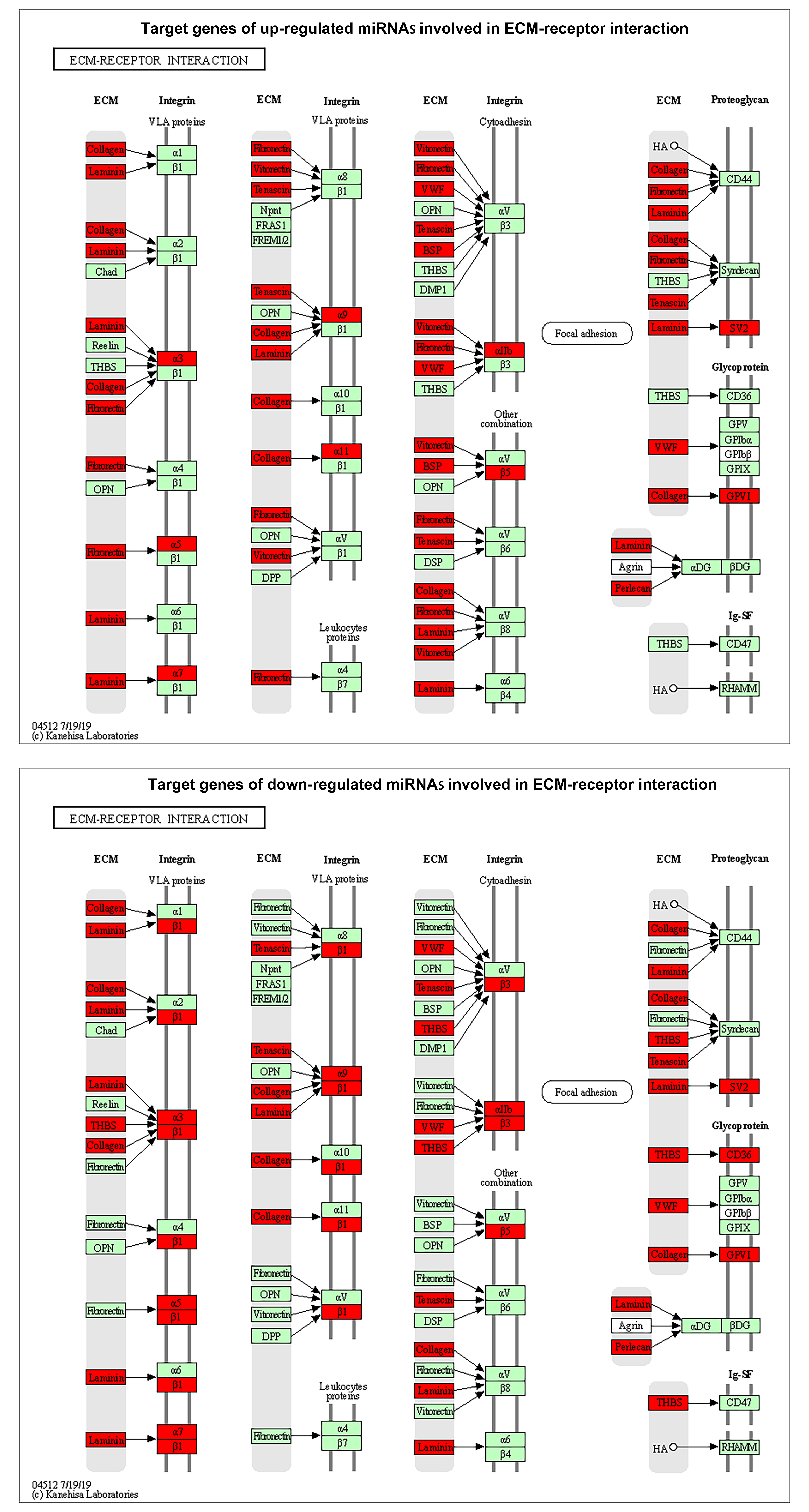

Supplement: Supplementary file 3 — Additional file 3: Figure S3. Details of DEmiRNAs’ target genes involved in ECM-receptor interaction in the fetus vs.child group. (A) Target genes of up-regulated DEmiRNAs. (B) Target genes of down-regulated DEmiRNAs. [file 12864_2021_7896_MOESM3_ESM.jpg]

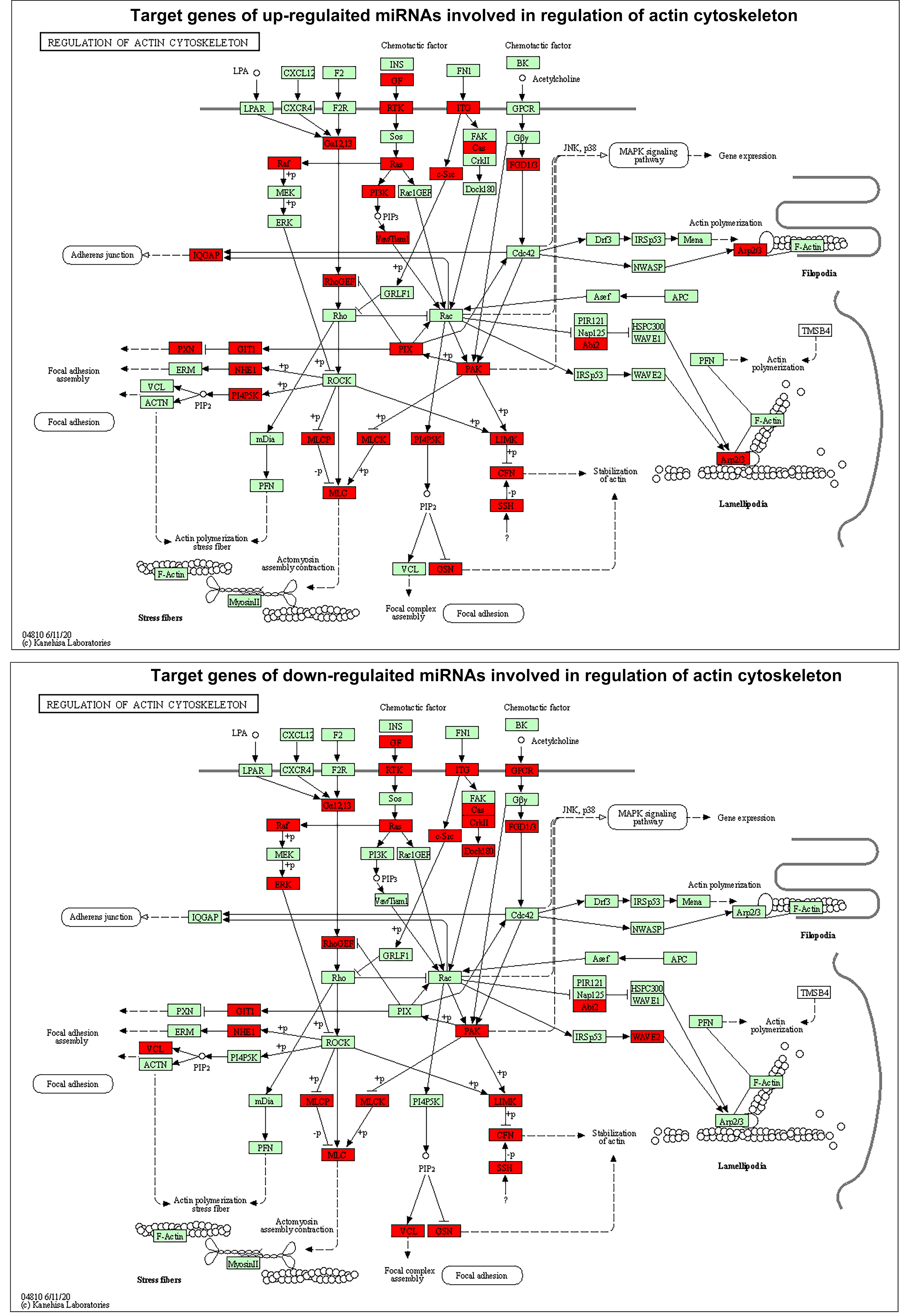

Supplement: Supplementary file 4 — Additional file 4: Figure S4. Details of DEmiRNAs’ target genes involved in regulation of actin cytoskeleton in the child vs. adult group. (A) Target genes of up-regulated DEmiRNAs. (B) Target genes of down-regulated DEmiRNAs. [file 12864_2021_7896_MOESM4_ESM.jpg]
